# Height outcomes in Korean children with idiopathic short stature receiving growth hormone treatment

**DOI:** 10.3389/fendo.2022.925102

**Published:** 2022-09-07

**Authors:** Hyun Wook Chae, Il-Tae Hwang, Ji-Eun Lee, Cheol Hwan So, Young-Jun Rhie, Jung Sub Lim, Eun Byul Kwon, Kyung Hee Yi, Eun Young Kim, Chae-Ku Jo, Kye Shik Shim, Ha-Yeong Gil, Min-Jeong Seong, Chung Mo Nam, Ji-Su Moon, Jin Soon Hwang

**Affiliations:** ^1^ Department of Pediatrics, Gangnam Severance Hospital, Yonsei University College of Medicine, Seoul, South Korea; ^2^ Department of Pediatrics, Hallym University Kangdong Sacred Heart Hospital, Seoul, South Korea; ^3^ Department of Pediatrics, Inha University Hospital, Inha University College of Medicine, Incheon, South Korea; ^4^ Department of Pediatrics, Wonkwang University Hospital, Jeollabuk-do, South Korea; ^5^ Department of Pediatrics, Korea University Ansan Hospital, Gyeonggi-do, South Korea; ^6^ Department of Pediatrics, Korea Cancer Center Hospital, Seoul, South Korea; ^7^ Department of Pediatrics, Hallym University Dongtan Sacred Heart Hospital, Hwaseong-si, South Korea; ^8^ Department of Pediatrics, Wonkwang University Sanbon Medical Center, Gyeonggi-do, South Korea; ^9^ Department of Pediatrics, Chosun University Hospital, Gwangju, South Korea; ^10^ Department of Pediatrics, Dong-A University College of Medicine, Busan, South Korea; ^11^ Department of Pediatrics, Kyung Hee University Hospital at Gangdong, Seoul, South Korea; ^12^ Medical Research Project Team, Internal Medicine (IM) Medical, Pfizer Korea, Seoul, South Korea; ^13^ Rare Disease, Medical Affairs, Pfizer Korea, Seoul, South Korea; ^14^ Department of Preventive Medicine, Yonsei University College of Medicine, Seoul, South Korea; ^15^ Division of Biostatistics, Department of Biomedical Systems Informatics, Yonsei University College of Medicine, Seoul, South Korea; ^16^ Department of Pediatrics, Ajou University Hospital, Gyeonggi-do, South Korea

**Keywords:** ISS (idiopathic short stature), growth hormone, height velocity, Korean ISS (idiopathic short stature) patients, dose of growth hormone, GH device type

## Abstract

**Objectives:**

Growth hormone (GH) therapy’s capacity to increase height velocity and height at the end of the study in children with idiopathic short stature (ISS) is controversial. We aimed to investigate the height standard deviation score (SDS) and height velocity of patients with ISS in Korea who received GH treatment.

**Methods:**

We retrospectively reviewed and performed linear mixed model and survival analyses on data from 12 tertiary hospitals in Korea, including subjects diagnosed with ISS from January 2009 to September 2019, treated with GH therapy for more than 6 months, and who were at a pre-pubertal state at the time of diagnosis.

**Results:**

We included 578 children (330 boys and 248 girls). The mean daily dose of GH in this study was 0.051 mg/kg, which was lower than the approved dose in Korea of 0.062 - 0.067 mg/kg. Height SDS was higher in patients who started treatment before the age of 6 years. The probability of reaching the target SDS (-1 SDS) from the beginning of treatment to 2–3 years after its start was higher in children starting treatment before the age of 6 years. The hazard ratio to reach the target SDS (-1 SDS) when using automatic pen or electronic devices was 1.727 times higher than that when using the needle and syringe device.

**Conclusion:**

ISS patients should start GH treatment at an early age, and even lower-than-recommended drug doses may be effective. The selection of automatic pen or electronic device can have a positive effect on reaching the target height SDS.

## Introduction

Idiopathic short stature (ISS) is generally defined as a condition in which height is not within the normal range and is below –2.0 standard deviation score (SDS) for the corresponding age and gender in a population without endocrinologic, nutritional, or chromosomal abnormalities (1–4). ISS differs from growth hormone deficiency (GHD) in that peak growth hormone levels are normal (5). Although the causes of short stature have been identified in some children, about 60%–80% of these children are considered as ISS without a definitive diagnosis (1, 2, 4, 6–11).

Recombinant human growth hormone (rhGH) was first approved in the United States in 1985 for the treatment of pediatric GHD and has been used worldwide since (4). The indications for GH treatment have gradually extended from GHD to non-GH-deficient short stature, such as Turner syndrome, chronic renal insufficiency, short children born small for gestational age, Noonan syndrome, and ISS (4, 12). The use of rhGH for ISS was approved in United States in 2003, and in Korea in 2009, while it has not been approved in Europe and Japan (13–18). In Korea, GH treatment for ISS patients is not covered by public health insurance, so the treatment is considered as the 2nd option due to its high cost (2, 4, 5, 19, 20).

Since the heterogeneous nature of nature of the ISS patient population, suggesting multiple potential pathophysiological mechanisms, it remains difficult to estimate the efficacy of GH for ISS patients worldwide, including that in Korea. Additionally, research results on the effects of the appropriate dosage of GH treatment for ISS patients and factors affecting the growth rate of children with ISS are still controversial (6, 15, 19, 21–24).

Therefore, we aimed to investigate the characteristics and treatment pattern of patients with ISS in Korea who received GH treatment, as well as identify the factors affecting their height SDS and height velocity.

## Subjects and methods

Data were collected through the retrospective review of medical records from 12 nationwide tertiary hospitals in Korea from 2019 to 2021. Hospitals participating in this study represent hospitals that treat and manage patients with ISS in Korea, judging from their geographical distribution, number of patients treated, and clinical experience and academic achievements of physicians. Before patient enrollment, the study protocol was approved by the 12 institutional review boards of participating institutions. (Approval number: AJIRB-MED-OBS-19-395)

As all patients who met the inclusion and exclusion criteria were enrolled during the observation period, a separate sample size calculation was not required. Patients who were diagnosed with ISS between January 2009 and September 2019, those who were treated for more than 6 months with any GH product prescribed in Korea, and those who were at prepubertal stage during diagnosis were enrolled. ISS was defined using a practical definition, that is, height below the 3rd percentile in the absence of any endocrine, metabolic, or other disease that might explain the short stature according to the investigator. In addition, we enrolled all patients with ISS who underwent GH provocation test or combined pituitary function test and other tests during hospitalization. After the start of GH treatment, patients without additional measurement of height or with chromosomal abnormalities, malnutrition, growth disorders, or low birth weight were excluded from the study.

A total of 578 children (330 boys and 248 girls) who started GH treatment between the ages of 4 and 14 years were included in the analysis. We collected the ISS diagnosis date, treatment start date, birth week, chronological age, bone age, height, weight, parental height, insulin like growth factor 1 (IGF-1), IGF binding protein 3 (IGFBP-3), and treatment pattern of subjects. Descriptive analysis was performed to identify the baseline characteristics of study subjects. Continuous variables were reported as mean and standard deviation and categorical variables were reported as frequency and proportion. SDS was calculated as follows: SDS = (patient’s measured value − mean value for age and sex − matched normal subjects)/(SD of the values for age and sex-matched normal subjects).

The normality of data was checked using the Shapiro–Wilk test. To compare treatment outcomes (baseline vs. treatment period), we conducted McNemar’s test and paired *t*-test or Wilcoxon’s signed-rank test according to the normality of data. Linear mixed model analysis was performed to identify factors affecting the height outcome of children with ISS. Survival analysis was performed to identify the variables affecting patients’ achievement of target height SDS (-1 SDS). All statistical analyses were performed using SAS 9.4 (SAS Inc., Cary, NC, USA).

## Results

### Children’s baseline characteristics

At the start of the treatment, the children’s chronological age was 7.54 ± 2.43 years and bone age was 6.08 ± 2.55 years. The mean height SDS of boys was −2.48 ± 0.57 and that of girls was −2.58 ± 0.73. The mean weight SDS of boys was −1.95 ± 0.84 and that of girls was −1.92 ± 0.93 (**Table 1**). The mean peak GH level of children was in the normal range (18.421 ± 7.90 ng/ml) and IGF-1 SDS was −0.60, which was lower than that of peers with the same age and gender (Table 1). The proportion of patients who used each device for GH treatment was similar between needle and syringe, automatic pen, and electronic devices (**Table 1**).

**Table 1:** Baseline characteristics before treatment.

	Total(n = 578)*		Boys(n = 330)*		Girls(n = 248)*		
	Mean	sd	Mean	sd	Mean	sd	p-value
Birth week (week)	38.72	2.06	38.71	2.09	38.74	2.03	0.9899
Birth weight (kg)	3.03	0.40	3.10	0.42	2.94	0.36	<.0001
Midparental height	163.87	7.22	169.34	3.57	156.65	3.53	<.0001
Peak GH (ng/ml)	18.42	7.90	18.47	7.65	18.36	8.24	0.4289
Baseline age (year)	7.54	2.43	7.56	2.59	7.52	2.21	0.7136
Height (cm)	112.13	12.95	112.58	13.53	111.54	12.14	0.5413
Height SDS	−2.52	0.65	−2.48	0.57	−2.58	0.73	0.0029
Weight (kg)	20.26	5.93	20.62	6.50	19.77	5.04	0.3426
Weight SDS	−1.93	0.87	−1.95	0.84	−1.92	0.93	0.6223
BMI (SDS)	−0.59	1.00	−0.62	1.02	−0.54	0.98	0.3090
Bone age (year)	6.08	2.55	5.90	2.58	6.33	2.50	0.0270
Bone Age − Chronological Age (year)	–1.44	1.04	−1.64	1.01	−1.16	1.03	<.0001
IGF-1 SDS	−0.60	0.94	−0.56	0.98	−0.65	0.89	0.4421
IGFBP-3 (SDS)	−0.02	2.09	0.09	2.04	−0.17	2.16	0.0991
GH dose (mg/kg)	0.051	0.077	0.051	0.076	0.052	0.080	0.5620
Treatment device	Rate (%)		Rate (%)		Rate (%)		0.7548
Needle & Syringe	50.35		50.91		49.60		
Automatic Pen or Electronic Device	49.65		49.09		50.40		

Baseline = Start of treatment.

Midparental height = Mothers height + Fathers height)/2 ± 6.5 cm.

GH dose (mg/kg): Dosage of GH per day/weight(kg).

*The sample size may be different for each variable.

p-value obtained using Wilcoxon’s signed-rank test according to the normality test.

### Effect of GH therapy on height outcomes

When treatment was continued, the children’s height SDS increased, and this change was statistically significant compared to the baseline height SDS. For boys, the height SDS measured at the start of treatment was −2.48 ± 0.57, and the height SDS after 3 years of treatment was −1.02 ± 0.81. The girls’ height SDS showed a similar trend to that of boys (the girl’s height SDS measured at the start of treatment was −2.58 ± 0.73, and that measured after 3 years was −1.12 ± 0.79) (**Tables  2**, **3**).

**Table 2:** Comparison of treatment outcomes: Boys.

Treatment period (year)	Baseline(n = 330)*	Treatment period ≤ 1(n = 330)*		1 < Treatment period ≤ 2(n = 218)*		2 < Treatment period ≤ 3(n = 99)*		Treatment period > 3(n = 62)*	
	Mean (sd)	Mean (sd)	p-value**	Mean (sd)	p-value**	Mean (sd)	p-value**	Mean (sd)	p-value**
Age (year)	7.56(2.59)	8.02(2.56)	<.0001	8.35(2.25)	<.0001	9.24(2.42)	<.0001	10.56(2.44)	<.0001
Height SDS	−2.48(0.57)	−1.93(0.58)	<.0001	−1.42(0.62)	<.0001	−1.11(0.69)	<.0001	−1.02(0.81)	<.0001
Weight SDS	−1.95(0.84)	−1.56(0.73)	<.0001	−1.18(0.75)	<.0001	−0.93(0.82)	<.0001	−0.75(0.91)	<.0001
BMI SDS	−0.62(1.02)	−0.64(0.82)	0.005	−0.59(0.82)	0.0001	−0.53(0.87)	0.016	−0.37(0.93)	0.4256
Bone age (year)	5.90(2.58)	6.32(2.54)	<.0001	7.17(2.59)	<.0001	8.50(2.72)	<.0001	10.09(2.76)	<.0001
Bone Age − Chronological Age (year)	−1.64(1.01)	−1.39(1.03)	0.0002	−1.72(1.05)	<.0001	−0.79(0.97)	<.0001	−0.54(1.19)	<.0001
IGF-1 (SDS)	−0.56(0.98)	0.62(1.28)	<.0001	0.97(1.4)	<.0001	1.4(1.79)	<.0001	1.34(1.67)	<.0001
IGFBP-3 (SDS)	0.09(2.04)	2.78(2.66)	<.0001	3.27(2.88)	<.0001	3.00(3.17)	<.0001	1.79(3.23)	0.0078
GH dose (mg/kg)	0.05(0.08)	0.05(0.06)	<0.0001	0.04(0.06)	0.0463	0.04(0.01)	0.5919	0.05(0.07)	0.4867
Treatment device	Rate (%)	Rate (%)	0.2568	Rate (%)	0.2059	Rate (%)	0.5637	Rate (%)	0.6547
Needle & Syringe	50.91	51.67		55.24		53.26		61.22	
Automatic Pen or Electronic Device	49.09	48.33		44.76		46.74		38.78	

* The sample size may be different for each variable.

** p-value: compared with baseline.

**Table 3:** Comparison of treatment outcomes: Girls.

Treatment period (year)	Baseline(n = 248)*	Treatment period ≤ 1 (n = 248) *		1 < Treatment period ≤ 2 (n = 162)*		2 < Treatment period ≤ 3 (n = 88)*		Treatment period > 3 (n = 45)*	
	Mean (sd)	Mean (sd)	p-value**	Mean (sd)	p-value**	Mean (sd)	p-value**	Mean (sd)	p-value**
Age (year)	7.52 (2.21)	7.98 (2.18)	<.0001	8.44 (2.06)	<.0001	8.92 (1.97)	<.0001	10.03 (1.78)	<.0001
Height SDS	−2.58 (0.73)	−2.04 (0.69)	<.0001	−1.59 (0.66)	<.0001	−1.25 (0.69)	<.0001	−1.12 (0.79)	<.0001
Weight SDS	−1.92 (0.93)	−1.62 (0.84)	<.0001	−1.28 (0.83)	<.0001	−1.02 (0.84)	<.0001	−0.97 (0.89)	<.0001
BMI SDS	−0.54 (0.98)	−0.64 (0.86)	<.0001	−0.59 (0.84)	<.0001	−0.52 (0.89)	0.0029	−0.57 (0.8)	0.0754
Bone age (year)	6.33 (2.5)	7.23 (2.49)	<.0001	7.78 (2.41)	<.0001	8.93 (2.34)	<.0001	10.15 (1.83)	<.0001
Bone age − Chronological age (year)	−1.16 (1.03)	−0.67 (1.02)	<.0001	−0.49 (1.00)	<.0001	−0.17 (1.08)	<.0001	0.14 (0.93)	<.0001
IGF-1 (SDS)	−0.65 (0.89)	0.53 (1.14)	<.0001	0.61 (1.09)	<.0001	0.65 (1.1)	<.0001	0.20 (1.03)	<.0001
IGFBP-3 (SDS)	−0.17 (2.16)	2.30 (2.78)	<.0001	2.97 (2.55)	<.0001	2.66 (2.99)	0.0002	2.02 (2.82)	0.1444
GH dose (mg/kg)	0.05 (0.08)	0.05 (0.06)	<0.0001	0.05 (0.05)	0.192	0.05 (0.06)	0.0511	0.05 (0.05)	0.002
Treatment device	Rate (%)	Rate (%)	0.5271	Rate (%)	0.0143	Rate (%)	0.1025	Rate (%)	0.3173
Needle & syringe	49.6	48.57		50.00		54.22		53.66	
Automatic pen or electronic device	50.4	51.43		50.00		45.78		46.34	

* The sample size may be different for each variable.

** p-value: compared with baseline.

**Figure 1:**
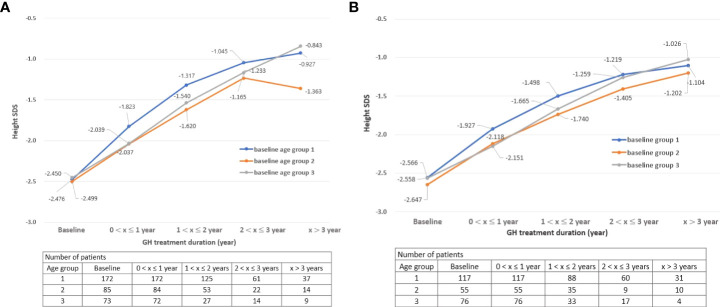
**(A)** Change in height SDS during treatment by age group: Boys. **(B)** Change in height SDS during treatment by age group: Girls. SDS, standard deviation score “x” denotes the duration of GH treatment (e.g., a value of 0 on the x-axis is the start time of GH treatment).

For both boys and girls, the group starting treatment before 6 years of age had the highest height SDS among the three groups (group 1: baseline age ≤ 6; group 2: 7 ≤ baseline age ≤ 9 for boys, 7 ≤ baseline age ≤ 8 for girls; group 3: baseline age ≥ 10 for boys, baseline age ≥ 9 for girls) (**Figures 1A, B**). This may mean that it is important for GH treatment to be started early and at an appropriate time.

**Figure 2:**
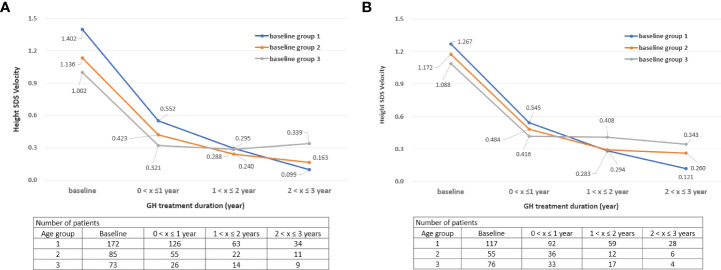
**(A)** Change in height SDS velocity during treatment by age group: Boys. **(B)** Change in height SDS velocity during treatment by age group: Girls. SDS, standard deviation score, “x” denotes the duration of GH treatment (e.g., a value of 0 on the x-axis is the start time of GH treatment).

The height velocity of children participating in this study up to 1 year after the start of GH treatment was 11.19 ± 5.24, and the average SDS of the height velocity was 1.22 ± 1.06. These results were similar to those of previous studies (height velocity of 6 months to 1 year treatment (cm/year): 7.57 ± 0.30 to 10.85 ± 2.34, growth velocity SDS: 1.47 ± 0.70) (4, 23, 25–27). Additionally, the height velocity was the highest in group 1 until 2 years of GH treatment, but the height velocity of group 3 was the highest after 3 years from the start of treatment. These results may have been influenced by puberty in the group 3, who started treatment at a relatively old age (**Figures 2A, B**).

### Factors affecting height outcomes

As a result of multivariate analyses, the height SDS of boys was 0.224 lower than that of girls (p = 0.002), and the height SDS was as high as 0.685 when treatment was continued for 3 years or longer compared to when the treatment period was less than 1 year (p < 0.001) (**Table 4**).

**Table 4:** Multivariate analysis (linear mixed model): ALL.

Dependant variable: Height SDS			
	Coef	SE	p-value
Intercept	−3.207	0.865	0.0002
Time-fixed covariates*			
**Gender**			
Girls	Ref	.	.
Boys	−0.224	0.069	0.0015
**Baseline age (year)**			
Baseline age ≤6	Ref	.	.
7 < Baseline age ≤ 9 boys 7 < Baseline age ≤ 8 girls	−0.187	0.056	0.0011
Baseline age ≥10 boys Baseline age ≥9 girls	−0.380	0.090	<.0001
Birth week (week)	0.005	0.010	0.6456
Bone age (year)	0.039	0.014	0.0060
Baseline height SDS	0.776	0.035	<.0001
Birth weight (kg)	0.045	0.052	0.3863
Midparental height	0.017	0.005	0.0007
Peak GH (ng/ml)	0.001	0.002	0.5635
Time-varying covariates**			
**Treatment Period (year)**			
Baseline<treatment period ≤ 1	Ref	.	.
1 < Treatment period ≤ 2	0.350	0.044	<.0001
2 < Treatment period ≤ 3	0.671	0.064	<.0001
Treament period > 3	0.685	0.092	<.0001
**Treatment device type**			
Needle & syringe type	Ref	.	.
Automatic pen type or electronic device type	0.024	0.035	0.4915
**GH dose (mg/kg)**	0.075	0.304	0.8044
**IGF-1 SDS**	0.121	0.015	<.0001
**IGFBP-3 SDS**	0.026	0.006	<.0001

GH dose (mg/kg) = dosage of GH per day/weight (kg).

* time-fixed covariate: only measured at baseline.

** time-varying covariate: measured at baseline and follow-up time.

Variables using value of previous visit: treatment device type, GH dose, bone age, IGF-1, IGFBP-3.

For boys, when treatment was started after the age of 10, the height SDS was 0.429 times lower than that when treatment was started before the age of 6 years (p = 0.001). The height SDS increased the most when treatment was maintained for 2 to 3 years (p < 0.001), and for girls, the height SDS value increased the most when treatment was continued for 3 years or longer (p < 0.001) (**Tables 5**, **6**).

**Table 5:** Multivariate analysis (linear mixed model): Boys.

Dependant variable: Height SDS			
	Coef	SE	p-value
Intercept	−2.281	1.153	0.0495
Time-fixed covariates*			
**Baseline age**			
Baseline age ≤ 6	Ref	.	.
7 ≤ Baseline age ≤ 9	−0.267	0.070	0.0003
Baseline age ≥ 10	−0.429	0.127	0.0011
Birth week (week)	0.013	0.013	0.3098
Bone age (year)	0.034	0.018	0.0632
Baseline height SDS	0.786	0.046	<.0001
Birth weight (kg)	0.051	0.063	0.4160
Midparental height	0.009	0.006	0.1585
Peak GH (ng/ml)	0.003	0.003	0.2291
Time-varying covariates**			
**Treatment period (year)**			
Baseline < treatment period ≤ 1	Ref	.	.
1 < Treatment period ≤ 2	0.325	0.058	<.0001
2 < Treatment period ≤ 3	0.654	0.081	<.0001
Treament period >3	0.463	0.138	0.0012
**Treatment device**			
Needle & syringe	Ref	.	.
Automatic pen or electronic device	0.027	0.043	0.5270
**GH dose (mg/kg)**	−2.321	2.639	0.3819
**IGF-1 SDS**	0.084	0.019	<.0001
**IGFBP-3 SDS**	0.028	0.008	0.0009

GH dose (mg/kg) = Dosage of GH per day/weight (kg).

* time-fixed covariate: only measured at baseline.

** time-varying covariate: measured at baseline and follow-up time.

Variables using value of previous visit: treatment device type, GH dose, bone age, IGF-1, IGFBP-3.

**Table 6:** Multivariate analysis (linear mixed model): Girls.

Dependant variable: Height SDS			
	Coef	SE	p-value
Intercept	−3.971	1.373	0.0045
Time-fixed covariates*			
**Baseline age**			
Baseline age ≤6	Ref	.	.
7 ≤ Basline age ≤ 8	−0.057	0.094	0.5486
Baseline age ≥9	−0.244	0.132	0.0696
Birth week (week)	−0.006	0.015	0.6937
Bone age (year)	0.033	0.022	0.1380
Baseline height SDS	0.764	0.054	<.0001
Birth weight (kg)	0.060	0.091	0.5099
Midparental height	0.024	0.007	0.0025
Peak GH (ng/ml)	0.002	0.004	0.6197
Time-varying covariates**			
**Treatment period (year)**			
Baseline < treatment period ≤ 1	Ref	.	.
1 < Treatment period ≤ 2	0.413	0.069	<.0001
2 < Treatment period ≤ 3	0.769	0.104	<.0001
Treament period > 3	0.897	0.134	<.0001
**Treatment device**			
Needle & syringe	Ref	.	.
Automatic pen or electronic device	0.006	0.056	0.9204
**GH dose (mg/kg)**	0.100	0.329	0.7625
**IGF-1 SDS**	0.177	0.023	<.0001
**IGFBP-3 SDS**	0.024	0.010	0.0236

GH dose (mg/kg) = Dosage of GH per day/weight (kg).

* time-fixed covariate: only measured at baseline.

** time-varying covariate: measured at baseline and follow-up time.

Variables using value of previous visit: treatment device type, GH dose, bone age, IGF-1, IGFBP-3.

The hazard ratio to reach the target SDS (-1 SDS) when using the automatic pen type or electronic device type is 1.727 times higher than that when using the needle and syringe device (**Appendix Table 1**). Furthermore, this result was more pronounced in boys (hazard ratio: 2.092, Appendix Tables 1-1, 1-2).

**Figure 3:**
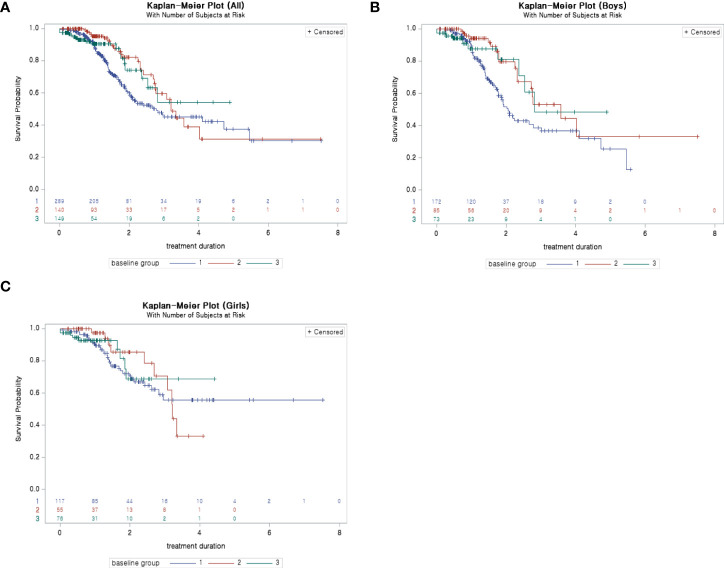
**(A)** Kaplan–Meier plot (survival probability: probability of not reaching −1 SDS): ALL. **(B)** Kaplan–Meier plot (survival probability: probability of not reaching −1 SDS): boys. **(C)** Kaplan–Meier plot (survival probability: probability of not reaching −1 SDS): girls. SDS, standard deviation score.

According to Kaplan–Meier plots, the probability of reaching the target SDS (-1 SDS) from the beginning of treatment to 2–3 years after the start of treatment for children who started treatment before 6 years of age was higher than that for children who started treatment after the age of 7 (**Figure 3**).

## Discussion

This was the first multi-center 10-year study on Korean pediatric patients with ISS to identify clinical characteristics and patterns of GH treatment and determine factors associated with height outcomes. We found that the height at the end of this study SDS is higher when GH treatment starts at an early age and when administered for 3 years or more, as opposed to less than 1 year. The average peak GH at baseline of children in this study was 18.42 (ng/ml), showing the characteristics of ISS pediatric patients with normal GH levels. This was consistent with the results of previous studies, in which the peak GH values of subjects at the time of ISS diagnosis were above 10 ng/ml, which were within a normal range (12.01–20.90 ng/ml) (21, 28). Regarding the administered dose, the mean daily dose in this study was 0.051 mg/kg, which was lower than the GH dose with regulatory approval of 0.062 - 0.067 mg/kg for patients with ISS in Korea.

From an efficacy point of view, the group who started before 6 years of age had the highest height SDS results after GH treatment for both boys and girls. There was a difference in height velocity according to age at the start of GH treatment, and the height velocity SDS was the highest for children who started GH treatment before the age of 6 years until 2 years of GH treatment (treatment duration 1). These results suggest that the treatment start age has a significant effect on the height velocity. Moreover, the height SDS of children showed a tendency to steadily increase while treatment was continued, as shown in **Figures 1A, B**. The average height SDS of children included in this study at the start of treatment was −2.52 ± 0.65, which was similar to that in other previous studies (−2.72 to −2.17) (4, 5, 21, 23, 28, 29). When GH treatment had started, boys’ height SDS increased by 0.55 and girls’ height SDS increased by 0.54 within 1 year of starting the treatment. If GH treatment had been continued for more than 3 years, the height SDS at the 3^rd^ year increased by 1.46 compared to baseline in both boys and girls (height SDS, boys: −2.48–−1.02, girls: −2.58–−1.12). These results were consistent with those of previous studies in which the height SDS increased by 0.40 to 0.57 from 6 months to the 1^st^ year after GH treatment and by 0.96–1.05 at the 3^rd^ year (4, 5, 30–32).

When considering both GH dose and the efficacy results comprehensively, GH treatment efficacy in this study was enough compared with that of previous studies, although the average GH dose in this study was lower than the recommended GH treatment dose. This revealed that the GH dose administered in a Korean clinical setting is lower than the recommended dose (29, 30, 33, 34) and that effective treatment is possible even without prescribing a high dose of GH to children with ISS. Although the dose initially approved in Korea (0.067 mg/kg/day, 0.37 mg/kg/week, 0.40 mg/kg/week) is lower that the dose recommended in international guidelines (0.47 mg/kg/week), the phenomenon of administering less than the approved dose for patients with ISS was also seen in previous Korean studies by Nam HK et al. in 2019 and Jeong HR et al. in 2014, which showed significantly improved growth outcomes even with low doses (35–40). In this study, the age at which GH treatment was started, duration of treatment, midparental height, baseline height SDS, IGF-1 SDS, and IGFBP-3 SDS were factors that affected the height outcomes of children with ISS. Remarkably, these factors have been identified in previous studies. Although the midparental height and the pediatric patients’ height and hormone status at the time of treatment cannot be altered, there were definite factors that could affect growth outcomes. It is emphasized that obtaining an accurate and early diagnosis and the early initiation of treatment are very important factors for better growth outcomes in ISS patients (4, 5, 23, 41, 42). Moreover, an important factor influencing the achievement of the target SDS in ISS patients is medication adherence. Parents of ISS children may feel sadness, guilt, and frustration for administering drugs to their children with a needle syringe, so the appropriate device type may help increase dosing compliance (27, 32, 43, 44). Treatment devices with improved dosing convenience and reduced pain, such as automatic pen and electronic device, can be helpful toward reaching the target SDS (12, 18).

The results presented above have a number of limitations. First, due to the nature of the noninterventional, multicenter, retrospective study, we could not control the time of visit of patients with ISS equally and we could not divide the patients into prepubertal and postpubertal groups. Second, because this study did not collect information on whether the GH was administered correctly daily or weekly according to the approved regimen, it is difficult to confirm patient compliance. Third, this study was conducted from 2009 to 2019 using Korea’s basic definition of ISS, so using the ISS diagnosis method from a more diverse and updated perspective was difficult. In addition, patients diagnosed with ISS were recruited on the discretion of the physicians at each hospital at the time, so patients could not be recruited according to strict and identical diagnostic criteria. Fourth, owing to the retrospective nature of this study, patients with ISS could not be continuously followed up until adulthood. Finally, considering the lack of research on patients with ISS in Korea, the study results were compared with those of previous studies in other countries where the average adult height may differ substantially from those of ethnic Koreans.

ISS is still used as a diagnostic label today, however, it is not a definitive diagnosis. While clinical and hormonal diagnostic techniques remain important, it is the emergence of genetic investigations that have led to numerous molecular discoveries in ISS subjects. The issue now is when to do some of the genetic screening, more target single gene, gene panel and array or whole exome or whole genome testing. Clinical evaluation, hormonal investigation and genetic sequencing are required to assess ISS subjects and should complement each other to identify the pathogenesis in poorly growing patients including ISS (45–53).

Although there were some limitations, this study contributes to understanding the characteristics and determining the timing and method of GH treatment in Korean pediatric patients with ISS.

Additionally, as shown in **Figure 1A**, the height SDS of group 2 decreased at the 4^th^ year than at the 3^rd^ year after treatment, which may be influenced by various biases. First, since the number of patients who received GH for more than 3 years in this study was very small, the trend of the graph is easily changed by a small number of patient results. Second, the possibility that the patients’ compliance with GH decreased with long-term administration cannot be ignored. Third, some patients in group 2 entered puberty after 3 years of treatment and the influence of puberty may have affected the graph (31).

In conclusion, ISS remains heterogeneous. Evaluating short stature in children using history, physical and laboratory examinations, and imaging and genetic studies is critical. Identifying biomarkers of rhGH response is also crucial. This study suggests that treatment for ISS should be started at an early age and that even if GH is administered at a dose lower than the approved one, its therapeutic effect can be sufficient. We highlight that using the automatic pen or electronic device instead of needle and syringe might have a positive influence on treatment effects.

## Data availability statement

The datasets presented in this article are not readily available because No access to raw dataset is allowed other than the Pfizer employee in charge of this study and the statistical analysis team. Requests to access the datasets should be directed to https://www.pfizer.com/contact.

## Ethics statement

The studies involving human participants were reviewed and approved by IRB approval numbers of 12 hospitals: CHOSUN 2019-10-007 WKUH2019-10-018 KHNMC 2019-10-027 HDT 2019-10-005-001 DAUHIRB-19-223 WMCSB201910-82 2019AS0220 2019-10-020 KIRAMS 2019-10-007 3-2019-0276 KANGDONG 2019-10-004 AJIRB-MED-OBS-19-395. Written informed consent for participation was not provided by the participants’ legal guardians/next of kin because: Because this study is retrospective medical chart review. So, the study subjects’ informed consent forms are not required in Korea.

## Author contributions

JH reviewed the study design and data collection instruments, collected data, and reviewed the manuscript. H-WC collected data, reviewed, and revised the manuscript. I-TH collected data, critically reviewed the analysis results for important intellectual content and reviewed the manuscript. J-EL, CS, Y-JR, JL, EK, KY, EK, C-KJ, and KS collected data and reviewed the study results. CN reviewed statistical analysis plan, reviewed the analysis results, and reviewed the manuscript. J-SM designed statistical analysis plan, carried out the analysis, and reviewed the manuscript. H-YG conceptualized and designed the study, coordinated and supervised data collection, developed, reviewed, and revised the manuscript. M-JS coordinated with investigators, reviewed the study results, and revised the manuscript. All authors approved the final manuscript as submitted and agree to be accountable for all aspects of the work.

## Funding

This study received funding from Pfizer Pharmaceuticals Korea Ltd. The funder had the following involvement with the study: study design. All the data were available to the authors and the authors analyzed the data with academic statisticians independently.

## Conflict of interest

H-YG and M-JS are employees of Pfizer Pharmaceuticals Korea Ltd.

The remaining authors declare that the research was conducted in the absence of any commercial or financial relationships that could be construed as a potential conflict of interest.

The authors declare that this study received funding from Pfizer Pharmaceuticals Korea Ltd. The funder had the following involvement with the study: study design.

## Publisher’s note

All claims expressed in this article are solely those of the authors and do not necessarily represent those of their affiliated organizations, or those of the publisher, the editors and the reviewers. Any product that may be evaluated in this article, or claim that may be made by its manufacturer, is not guaranteed or endorsed by the publisher.

## Supplementary material

Appendix Figure 1
**(A)** Change in height SDS during treatment – boys. **(B)** Change in height SDS during treatment– girls “x” denotes the duration of GH treatment (e.g., a value of 0 on the x-axis is the start time of GH treatment).

Appendix Figure 2 **(A)** Change in height SDS velocity during treatment – boys. **(B)** Change in height SDS velocity during treatment – girls “x” denotes the duration of GH treatment (e.g., a value of 0 on the x-axis is the start time of GH treatment).
