# Supplementary figures and images for: Height outcomes in Korean children with idiopathic short stature receiving growth hormone treatment
Source: Front Endocrinol (Lausanne). 2022 Sep 7;13:925102. doi: 10.3389/fendo.2022.925102 (PMC9490583; doi:10.3389/fendo.2022.925102)

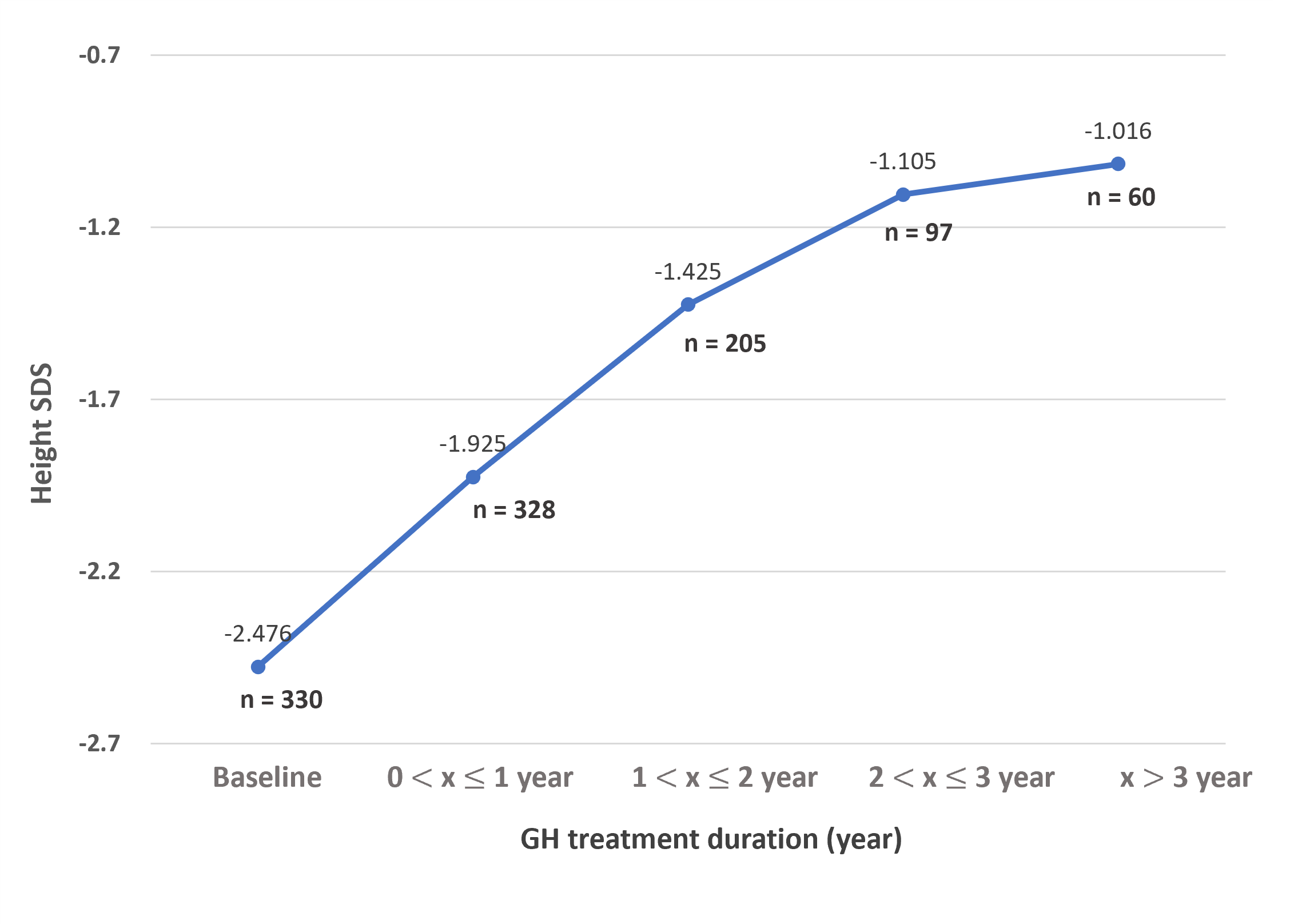

Supplement: Appendix Figure 1 — (A) Change in height SDS during treatment – boys. (B) Change in height SDS during treatment– girls “x” denotes the duration of GH treatment (e.g., a value of 0 on the x-axis is the start time of GH treatment). [file Image_1.tiff]

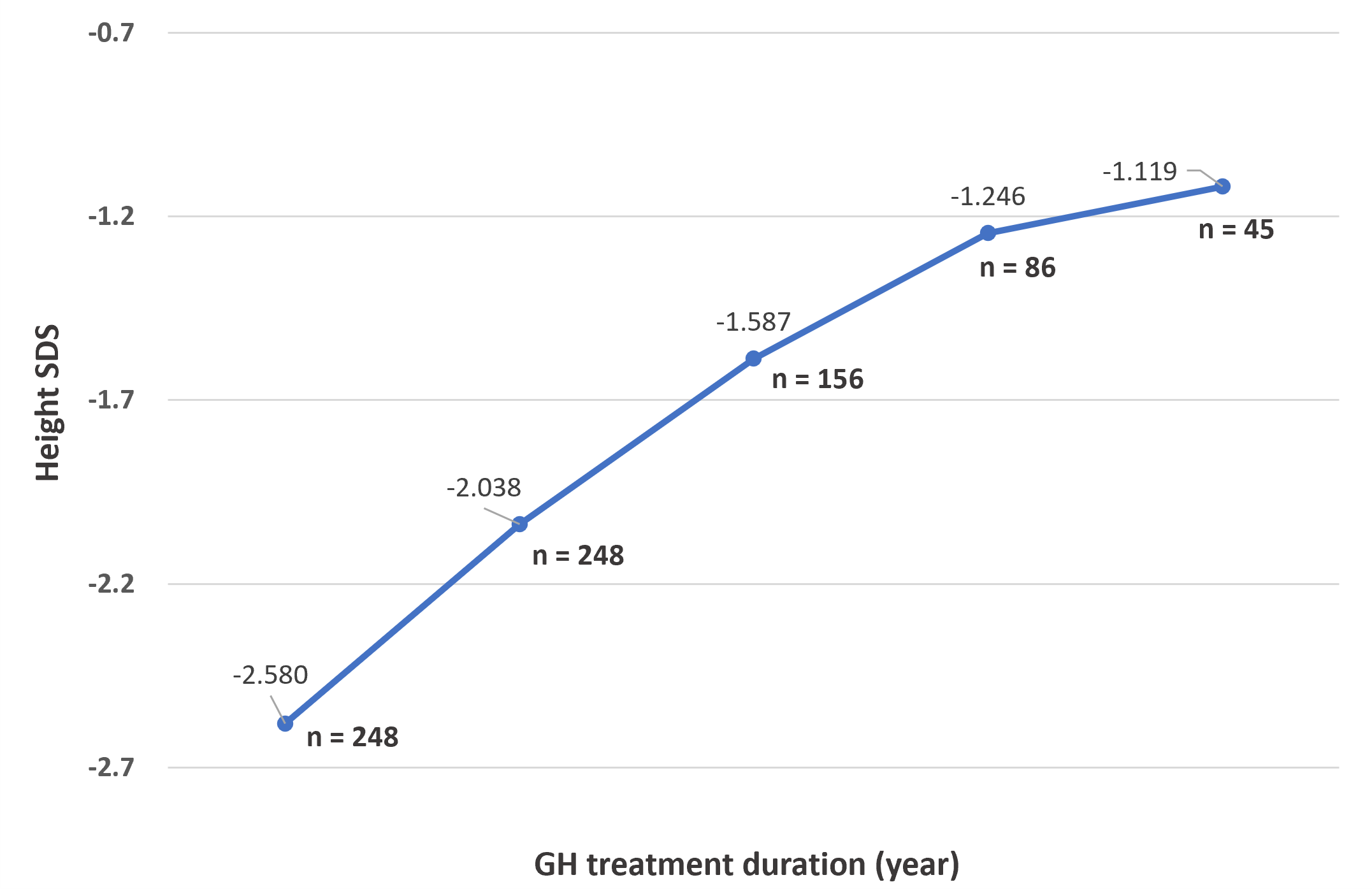

Supplement: Appendix Figure 2 — (A) Change in height SDS velocity during treatment – boys. (B) Change in height SDS velocity during treatment – girls “x” denotes the duration of GH treatment (e.g., a value of 0 on the x-axis is the start time of GH treatment). [file Image_2.tiff]

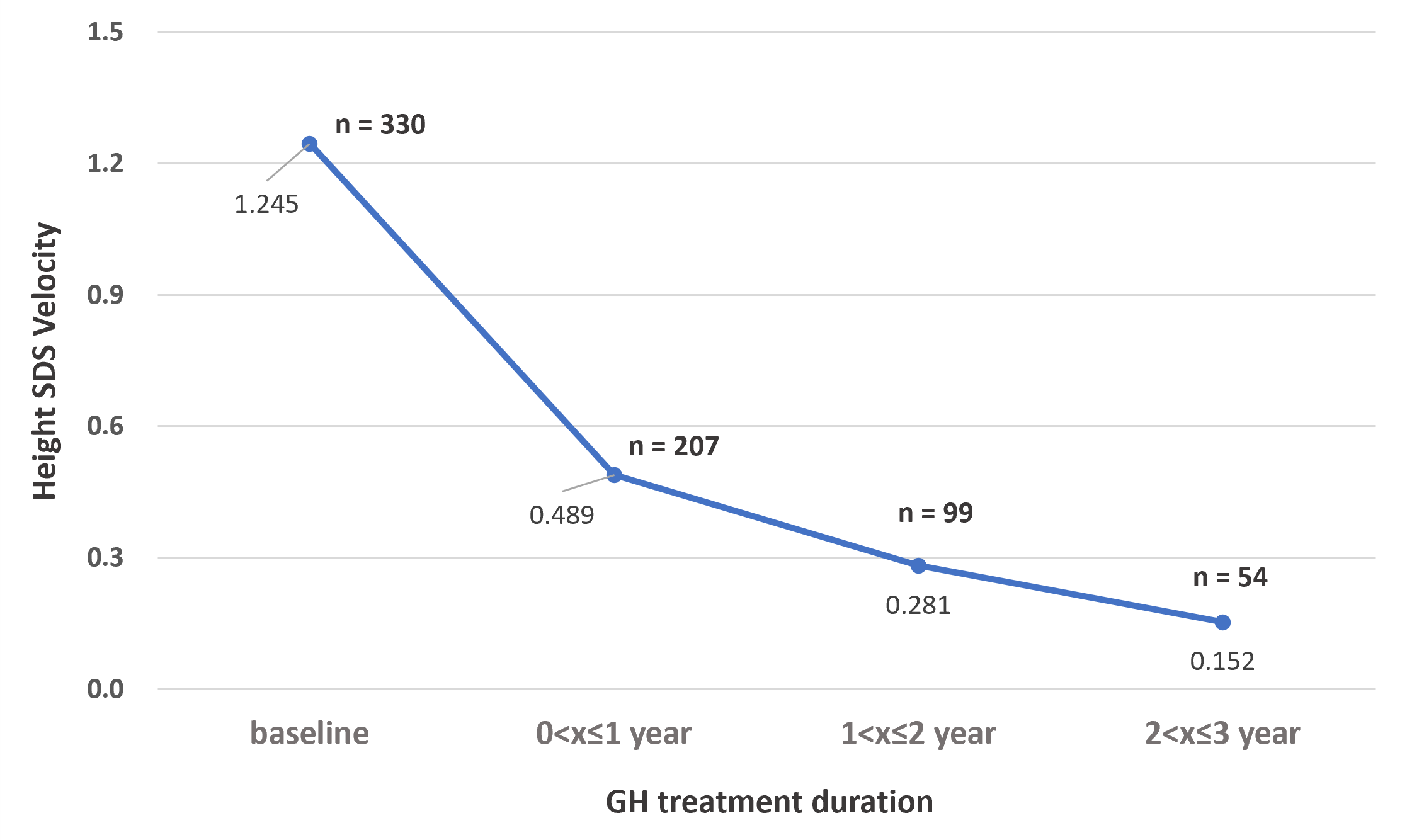

Supplement: Supplementary file 3 [file Image_3.tiff]

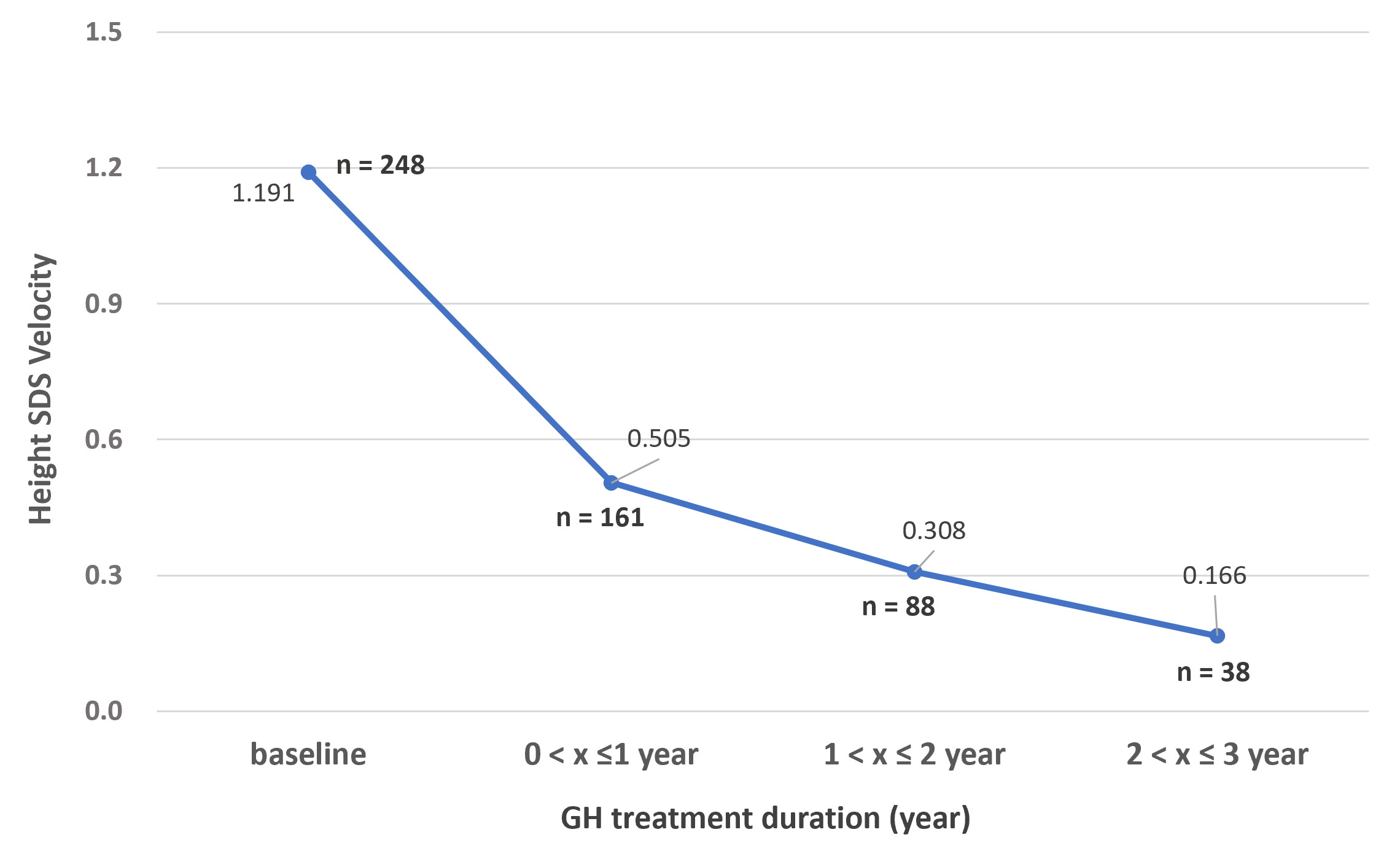

Supplement: Supplementary file 4 [file Image_4.tiff]
