# Supplementary material for: Height outcomes in Korean children with idiopathic short stature receiving growth hormone treatment
Source: Front Endocrinol (Lausanne). 2022 Sep 7;13:925102. doi: 10.3389/fendo.2022.925102 (PMC9490583; doi:10.3389/fendo.2022.925102)
Supplement: Supplementary file 5 [file Table_1.docx]

| **Appendix Table 1. Survival analysis (Cox regression) - ALL** | |  |  |  |  |  |
| --- | --- | --- | --- | --- | --- | --- |
|  | **Coef** | **SE** | **p-value** | **Hazard Ratio** | **95% Hazard Ratio  Confidence Limits** | |
| **Gender** |  |  |  |  |  |  |
| Girls | Ref. |  |  |  |  |  |
| Boys | 0.2057 | 0.4914 | 0.6756 | 1.2280 | 0.4690 | 3.2180 |
| **Baseline age** |  |  |  |  |  |  |
| Baseline age ≤ 6 | Ref |  |  |  |  |  |
| 7≤Baseline age≤9 Boys  7≤Baseline age≤8 Girls | -0.8697 | 0.3017 | 0.0039 | 0.4190 | 0.2320 | 0.7570 |
| Baseline age≥10 Boys  Baseline age≥9 Girls | -0.3451 | 0.3364 | 0.3049 | 0.7080 | 0.3660 | 1.3690 |
| **Baseline height SDS** | 2.2776 | 0.2478 | <.0001 | 9.7530 | 6.0010 | 15.8510 |
| **Birth weight (kg)** | -0.3077 | 0.2604 | 0.2374 | 0.7350 | 0.4410 | 1.2250 |
| **Midparental height** | 0.0303 | 0.0339 | 0.3717 | 1.0310 | 0.9640 | 1.1020 |
| **Treatment device type** |  |  |  |  |  |  |
| - Needle & Syringe Type | Ref |  |  |  |  |  |
| - Automatic Pen Type or Electronic device Type | 0.5464 | 0.2318 | 0.0184 | 1.7270 | 1.0960 | 2.7200 |
| **GH dose (mg/kg)** | 4.6203 | 1.6213 | 0.0044 | 1.0152$\times$10^2^ | 4.2310 | 2.4359$\times$10^3^ |
| GH dose (mg/kg) = Dosage of GH per day / weight (kg) |  |  |  |  |  |  |
| *time: treatment duration (year), event: - 1 SDS |  |  |  |  |  |  |
| Variables using value of previous visit: treatment device type, GH dose | | |  |  |  |  |
