# Supplementary material for: Height outcomes in Korean children with idiopathic short stature receiving growth hormone treatment
Source: Front Endocrinol (Lausanne). 2022 Sep 7;13:925102. doi: 10.3389/fendo.2022.925102 (PMC9490583; doi:10.3389/fendo.2022.925102)
Supplement: Supplementary file 6 [file Table_2.docx]

| **Appendix table 1-1. Survival analysis (Cox regression) - Boys** | |  |  |  |  |  |
| --- | --- | --- | --- | --- | --- | --- |
|  | **Coef** | **SE** | **p-value** | **Hazard Ratio** | **95% Hazard Ratio  Confidence Limits** | |
| **Baseline age** |  |  |  |  |  |  |
| Baseline age ≤ 6 | Ref |  |  |  |  |  |
| 7≤Baseline age≤9 | -1.2264 | 0.3936 | 0.0018 | 0.2930 | 0.1360 | 0.6340 |
| Baseline age≥10 | -0.1782 | 0.4587 | 0.6977 | 0.8370 | 0.3410 | 2.0560 |
| **Baseline height SDS** | 2.1830 | 0.3323 | <.0001 | 8.8730 | 4.6260 | 17.0190 |
| **Birth weight (kg)** | -0.3429 | 0.2966 | 0.2475 | 0.7100 | 0.3970 | 1.2690 |
| **Midparental height** | 0.0207 | 0.0431 | 0.6308 | 1.0210 | 0.9380 | 1.1110 |
| **Treatment device type** |  |  |  |  |  |  |
| - Needle & Syringe Type | Ref |  |  |  |  |  |
| - Automatic Pen Type or Electronic device Type | 0.7382 | 0.2836 | 0.0092 | 2.0920 | 1.2000 | 3.6480 |
| **GH dose (mg/kg)** | 0.7145 | 9.6999 | 0.9413 | 2.0430 | 0.0000 | 3.6883$\times$10^8^ |
| GH dose (mg/kg) = Dosage of GH per day / weight (kg) | |  |  |  |  |  |
| Variables using value of previous visit: treatment device type, GH dose | | |  |  |  |  |
