# Supplementary material for: Height outcomes in Korean children with idiopathic short stature receiving growth hormone treatment
Source: Front Endocrinol (Lausanne). 2022 Sep 7;13:925102. doi: 10.3389/fendo.2022.925102 (PMC9490583; doi:10.3389/fendo.2022.925102)
Supplement: Supplementary file 7 [file Table_3.docx]

| **Appendix Table 1-2. Survival analysis (Cox regression) - Girls** | |  |  |  |  |  |
| --- | --- | --- | --- | --- | --- | --- |
|  | **Coef** | **SE** | **p-value** | **Hazard Ratio** | **95% Hazard Ratio  Confidence Limits** | |
| **Baseline age** |  |  |  |  |  |  |
| Baseline age ≤ 6 | Ref. |  |  |  |  |  |
| 7≤Baseline age≤8 | -0.1104 | 0.5213 | 0.8324 | 0.8960 | 0.3220 | 2.4880 |
| Baseline age≥9 | -0.4141 | 0.5499 | 0.4515 | 0.6610 | 0.2250 | 1.9420 |
| **Baseline height SDS** | 2.9992 | 0.4642 | <.0001 | 20.0680 | 8.0790 | 49.8500 |
| **Birth weight (kg)** | 0.1916 | 0.5956 | 0.7477 | 1.2110 | 0.3770 | 3.8920 |
| **Midparental height** | 0.0873 | 0.0647 | 0.1772 | 1.0910 | 0.9610 | 1.2390 |
| **Treatment device type** |  |  |  |  |  |  |
| - Needle & Syringe Type | Ref. |  |  |  |  |  |
| - Automatic Pen Type or Electronic device Type | 0.1147 | 0.4290 | 0.7891 | 1.1220 | 0.4840 | 2.6000 |
| **GH dose (mg/kg)** | 5.5230 | 1.8230 | 0.0024 | 2.5038$\times$10^2^ | 7.0280 | 8.9203$\times$10^3^ |
| GH dose (mg/kg) = Dosage of GH per day / weight (kg) |  |  |  |  |  |  |
| Variables using value of previous visit: treatment device type, GH dose | | |  |  |  |  |
